# Flurbiprofen-Loaded Solid SNEDDS Preconcentrate for the Enhanced Solubility, In-Vitro Dissolution and Bioavailability in Rats

**DOI:** 10.3390/pharmaceutics10040247

**Published:** 2018-11-28

**Authors:** Rae Man Kim, Dong-Jin Jang, Yu Chul Kim, Jin-Ha Yoon, Kyoung Ah Min, Han-Joo Maeng, Kwan Hyung Cho

**Affiliations:** 1 College of Pharmacy and Inje Institute of Pharmaceutical Sciences and Research, Inje University, Gimhae 50834, Korea; didi032@naver.com (R.M.K.); minkahh@inje.ac.kr (K.A.M.); 2 Department of Pharmaceutical Engineering, Inje University, Gimhae 50834, Korea; djjang@inje.ac.kr (D.-J.J.); yckim@inje.ac.kr (Y.C.K.); 3 College of Pharmacy, Gachon University, Incheon 21936, Korea; jinha89@daum.net

**Keywords:** flurbiprofen, solid self-nanoemulsifying drug delivery system, dissolution, bioavailability

## Abstract

The aim of this work was to prepare and optimize a solid self-nanoemulsifying drug delivery system pre-concentrate (SSP) containing water-insoluble flurbiprofen (FL) using a novel pseudo-ternary phase diagram. The pseudo-ternary phase diagram, composed of FL as the drug and dispersion core, Kollisolv MCT 70 as the oil phase, and TPGS (tocopherol polyethylene glycol 1000 succinate) as the surfactant, was constructed for the determination of the SSP region. SSP was investigated in terms of particle size, physical state by differential scanning calorimetry (DSC) and powder X-ray diffraction (PXRD), in vitro dissolution and oral pharmacokinetics in rats. The determined SSP (FL/Kollisolv MCT 70/TPGS = 10/10/80, weight %) in the pseudo-ternary phase diagram had the melting point of 32.37 °C and uniform mean particle size of below 30 nm without any precipitation of FL in the dispersion. In the dissolution test, the SSP exhibited 95.70 ± 3.40% of release at 15 min, whereas the raw FL showed poor dissolution (i.e., 6.75 ± 1.30%) at that time point. In addition, the SSP showed the enhanced oral absorption (i.e., 1.93-fold increase in AUC_infinite_) as compared to the suspension group of raw FL. Therefore, the developed SSP would be a promising drug delivery system with excellent solubilization, dissolution, and bioavailability for FL.

## 1. Introduction

Flurbiprofen (FL) (2-(2-fluoro-4-biphenyl) propionic acid) is a cyclooxygenase (COX-1 and 2) inhibitor that blocks the synthesis of prostaglandin E2 and is reported to have analgesic and anti-inflammatory effects [1]. Its clinical efficacy has been known as symptom and pain relief in several inflammation diseases, such as rheumatoid arthritis, osteoarthritis, arthralgia, and ankylosing spondylitis [2]. This drug is classified to BCS class II and exhibits low aqueous solubility (5–10 μg/mL) [3,4]. Solubilization formulation influences the absorption and oral bioavailability of FL, since FL is practically water-insoluble [5]. Various formulations, such as solid lipid nanoparticles (SLNs), β-cyclodextrin complex, nanosuspensions, and a self-microemulsifying drug delivery system (SMEDDS), have been applied to improve bioavailability by increasing the solubility and dissolution rate of FL [6,7,8,9].

The self-nanoemulsifying drug delivery system (SNEDDS) is a formulation technology to be applied for poorly water-soluble drugs with high lipophilicity. Due to optimization, the SNEDDS formulation containing oils, and surfactants (or co-surfactants) enable an increase of the solubilization of drugs in the aqueous medium [10]. The SNEDDS are a particulate type of drug carriers with the size range of 20~200 nm dispersed in water; drug molecules are incorporated into the interfacial films or nano-sized oil droplets possessing a large interfacial surface area. With this approach, it is possible to enhance drug solubility and absorption in the gastrointestinal tract, thereby improving oral bioavailability. Representatively, some commercial drugs are available in the SNEDDS formulations, such as ritonavir (Norvir^®^), cyclosporine A (Sandimmune Neoral^®^), saquinavir (Fortovase^®^), and tipranavir (Aptivus^®^) [11,12,13]. However, for these formulations, several reports have demonstrated some hypersensitivity reactions as side effects, presumably related to types of surfactants. Cremophor EL has been known to cause the gastrointestinal tract toxicity [14]. Moreover, these conventional SNEDDS formulations have been developed in the liquid state, which imposed several limitations on the manufacturing process, such as high production costs and physical instability issues, including drug precipitation, phase separation, and/or the incompatibility between the fill ingredients and the gelatin capsule shell at the storage temperature [15].

On the other hand, in order to overcome formulation challenges associated with the liquid SNEDDS, the solid SNEDDS has been widely investigated for the drugs with poor water solubility. Commonly, the solid SNEDDS was prepared by adsorbing liquid SNEDDS materials onto solid carriers, such as carboxymethylcellulose sodium, polyvinylalcohol, silicon dioxide, and magnesium stearate, followed by the spray drying [16]. For example, representative drug compounds with low aqueous solubility (e.g., curcumin, dexibuprofen, and nimodipine) have been developed with the solid SNEDDS method, showing improved physical stability in the final products [17,18]. These studies suggest that the physical stability of the final drug formulations can be ensured by devising the solid SNEDDS; other advantages of the solid SNEDDS are that it provides enhanced drug solubility or bioavailability. The solid SNEDDS has been mainly prepared by dissolving the liquid state in a co-solvent system and further removing the solvent by the spray drying to get powdered products. However, despite the improved physical stability, this method still has several disadvantages, such as a complicated manufacturing process, the use of a large number of additives, and increased particle sizes after redispersion of solidified SNEDDS in water.

In the present study, a new pseudo-ternary phase diagram was constructed only using a solid surfactant, oil, and drug. Solid SNEDDS preconcentrate (SSP) was manufactured using the simple mixing method without employing solid carriers, showing small uniform particles in the dispersion, and the solid state region of SSP was confirmed in the pseudo-ternary phase diagram. The physical and chemical properties of SSP were investigated in terms of particle size measurement, differential scanning calorimetry (DSC), and powder X-ray diffractometer (PXRD). The optimized SSP was further investigated for dissolution performance and oral pharmacokinetics in rats.

## 2. Materials and Methods

### 2.1. Materials

FL was purchased from Kolon Life Science Inc. (Seoul, Korea). Kollisolv MCT 70 (medium chain capric triglyceride) and Kolliphor HS 15 (polyoxyl 15 hydroxystearate) were kindly provided by BASF (Ludwigshafen, Germany). Lauroglycol 90 (propylene glycol monolaurate, type), Capmul MCM C8 (mono/diglycerides of caprylic acid), and Gelucire 44/14 (lauroyl polyoxyl-32 glycerides) were kindly provided by Gattefosse (Saint-Priest, France). TPGS (tocopherol polyethylene glycol 1000 succinate) was purchased from ISOCHEM (Vert-le-Petit, France). All other chemicals were of reagent grade and were used without further purification.

### 2.2. HPLC Condition

The HPLC analysis of FL in samples was conducted using the Waters 2695 HPLC system (Waters, Milford, MA, USA), which is equipped with a UV-Vis detector (Waters 2487, Waters, Milford, MA, USA). FL was separated by a reverse-phase column (C18, 3.5 μm, 2.1 mm × 10 cm) (Shiseido, Tokyo, Japan). The mobile phase was a mixture of water, acetonitrile, and acetic acid (12:7:1, *v*/*v*/*v*). The HPLC analysis was performed with a flow rate of 0.5 mL/min. The injected volume of the sample was 20 μL, and UV detection was monitored at 254 nm. Data acquisition and processing were performed using the Waters LC Solution software (Em power 2.0 version).

### 2.3. Solubility Test

The solubility of FL in various oils and surfactants was evaluated. Kollisolv MCT 70, Lauroglycol 90, Capmul MCM C8, Gelucire 44/14, Kolliphor HS 15, or TPGS were commonly tested at 60 °C, considering the intrinsic melting point of solid surfactants. An excess of FL powder was added to 1 g of each oil and surfactant; thereafter, the solution was stirred until it was suspended. 0.5 g of supernatant was obtained after centrifuged at 15,000 rpm for 10 min and then diluted 5 times with the above-mentioned mobile solution under the HPLC condition specified above. The FL amount was measured thrice.

### 2.4. Characterization of Various Vehicle Compositions

In order to select specific oils and surfactants that would exhibit the optimum dispersed particle size, various vehicle compositions without the drug were prepared and evaluated. As shown in Table 1, oils and surfactants were weighed at various weight ratios (1/3, 2/2, or 3/1), heated to 60 °C, and stored at 4 °C for 24 h for possible solidification. The samples were taken out and equilibrated at room temperature. Then, 100 mg of the sample was taken, and 1 mL of water at 37 °C was added. This was followed by vortexing for 1 min to completely disperse the samples. The particle size of the samples was measured by particle size analyzer, and the melting point of each vehicle composition was measured by DSC.

### 2.5. Particle Size Measurement

**Figure 1:**
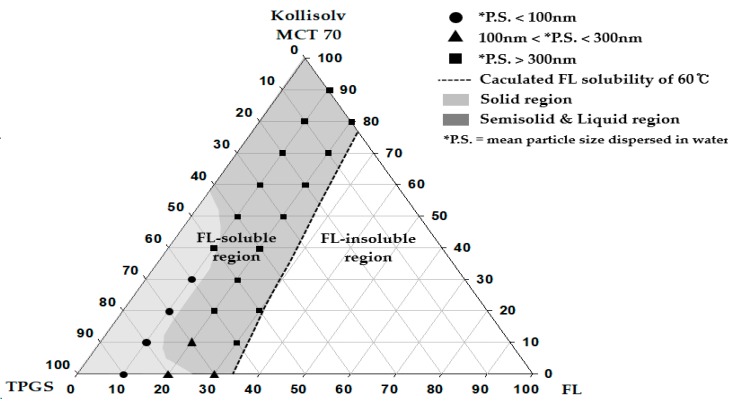
Pseudo-ternary phase diagram composed of flurbiprofen (FL), tocopherol polyethylene glycol 1000 succinate (TPGS), and Kollisolv MCT 70.

The mixture and formulation (a total of 100 mg), shown in Figure 1, and Table 1 and Table 2, was dispersed into 3 mL of water with a mild vortexing for 1 min. The particle size was determined using a particle size analyzer (NanoBrook 90Plus, Brookhaven instruments Corporation, Holtsville, NY, USA) at the wavelength of 659 nm and the scattering angle of 90°. The temperature was set at 25 °C, and the number of measurements was set at 5 cycles. For measurement, each sample was diluted 20 times with distilled water. The measurements were repeated thrice.

### 2.6. Differential Scanning Calorimetry (DSC)

The DSC was performed using a DSC Q20 (TA Instruments, New Castle, DE, USA). The device was calibrated using an Indium, and the samples were scanned under nitrogen gas purging (20 mL/min). For measurements, 3–5 mg of samples was placed in an aluminum pan and covered with an aluminum lid. The heating rate was set at 10 °C/min. The results of the analysis were assessed in the range of 10 °C~140 °C. The peak melting temperature for each endothermic curve in the thermogram was determined automatically using the DSC solution software (TA Universal Analysis 2000).

### 2.7. Construction of Pseudo-Ternary Phase Diagram

A pseudo-ternary phase diagram was constructed with FL, Kollisolv MCT 70, and TPGS. FL solubility (%) was calculated for the varied composition at the 10% weight ratio interval between Kollisolv MCT 70 and TPGS within the pseudo-ternary phase diagram. Thereafter, the composition of (*C*, *D*, *E*) determined by Equation (1) was plotted (see Figure 1). Equation (1) was used to calculate FL solubility (E, %) with the predetermined C and D.

FL solubility (E, %) at C and D = [(*A* × *C*) + (*B* × *D*)]/100, (1)

*A* = FL solubility (%) in Kollisolv MCT 70 from Table 3. *B* = FL solubility (%) in TPGS from Table 3.
*C* = predetermined composition (%) of Kollisolv MCT 70. *D* = predetermined composition (%) of TPGS. *C* + *D* + *E* = 100%.

In the next step, we prepared 22 formulations within the FL solubility curve (see Figure 1). FL was added to the mixture of oil and surfactant at 60 °C and completely solubilized and stored at 4 °C for 24 h. The properties of the samples and the particle size dispersed in water were then evaluated.

### 2.8. Powder X-ray Diffraction (PXRD)

*PXRD* was measured using an X-ray diffractometer (Rigaku, Ultima IV, Toyko, Japan), which is equipped with a Linxeye 1-D detector. Each sample was added to the grid, and the diffraction pattern of each sample was measured using a Cu/Kα radiation source (40 kV and 40 mA) with the acquisition time of 0.2 s per step. The scanning range was 2~50° in the 2θ range.

### 2.9. Dissolution Test

Dissolution tests of the FL-loaded formulations and raw FL were performed using a VK 7000 dissolution tester (VanKel, Cary, NC, USA). Each sample filled in a gelatin capsule shell (#1), equivalent to 40 mg FL, was prepared and place into the dissolution tester at 37 ± 0.5 °C using the paddle method of 50 rpm with 900 mL of pH 1.2 buffer. An aliquot (5 mL) of the samples was collected at the predetermined time intervals, filtered through a 0.45 um membrane filter (DISMIC^®^-13HP, ADVANTEC^®^, Tokyo, Japan), and diluted 2 times with diluent solution (water/ACN = 11/9, *v*/*v*). The amount of FL was determined by the HPLC analysis as described in the HPLC condition above.

### 2.10. Oral Pharmacokinetic Study in Rats

In the fasted male Sprague Dawley (SD) rats weighing 280–290 g (Orient Bio, Sungnam, Korea), the oral pharmacokinetics was investigated and compared for the FL suspension in 0.3% CMC-Na and F2 formulation (*n* = 4–5). All animal experiments were performed in accordance with the Guidelines for Animal Care and Use issued by Gachon University, as described previously [19]. The animals were fasted overnight, but allowed to drink water. Under the anesthetic condition with Zoletil (20 mg/kg, intramuscular injection), femoral arteries were cannulated for blood sample collection with a Clay Adams PE-50 polyethylene tube (Parsippany, NJ, USA) filled with heparinized saline (20 IU/mL). After their recovery from the surgery, the rats were orally administered FL at the dose of 10 mg/kg. After the oral administration, blood samples (100 µL) were collected at predetermined time intervals (0, 15, 30, 60, 90, 120, 180, 240, 360, 480, and 1440 min). Plasma was subsequently obtained by centrifuging whole blood at 4 °C for 10 min and then stored at −20 °C before the analysis. Plasma samples were pretreated with a methanolic solution containing internal standard (IS, 500 ng/mL), and then centrifuged at 12,000 g at 4 °C for 15 min to obtain the supernatant.

For the analysis of FL in rat plasma, we used the UHPLC system with the Agilent 1290 Infinity II UHPLC system (Agilent Technologies, Santa Clara, CA, USA), equipped with an auto-sampler (G7167B), a flexible pump (G7104A), a Multicolumn Thermostat (MCT) (G7116B), and a DAD detector (G7117A). A Synergi™ 4 µm polar, Reversed-Phase (RP) 80A column (150 × 2.0 mm, Phenomenex, Torrance, CA, USA) successfully achieved a good separation with endogenous substances. Isocratic condition was applied with the mobile phase consisting of potassium phosphate buffer (50 mM, pH 3.5) and acetonitrile (25:75, *v*/*v*) at the flow rate 0.2 mL/min. The injection volume was set at 2 µL, and the DAD detector to 248 nm. The temperature of the column and the autosampler tray was maintained at 25 °C and 4 °C, respectively.

### 2.11. Data Analysis

The pharmacokinetic parameters were calculated using the Pharsight^®^ WinNonlin program (Ver 8.0, Certara, Moutain View, CA, USA). The analyzed parameters included the maximum plasma concentration (*C*_max_), time to reach the maximum plasma concentration (*T*_max_), half-life (*T*_1/2_), area under the plasma concentration-time curve to the last time point or infinity time (AUC_last_ or AUC_∞_), and mean residence time (MRT). Briefly, AUC_last_ was calculated by the linear trapezoidal method from 0 to the last time point. AUC_∞_ was calculated as AUC_last_ + *C*_t_/*λ*, where *C*_t_ is the last measured concentration and *λ* denotes the slope of the terminal phase. Experiments were performed in triplicates and data were presented as mean ± standard deviation (SD). Student’s *t*-test or one-way ANOVA was used to determine statistically significant differences among groups. The Prism software (version 4.0) was used for statistical analyses. *p* < 0.05 was considered to be statistically significant.

## 3. Results and Discussion

### 3.1. Solubility Test

To determine the suitable surfactants or oil components for solubilizing FL in the SNEDDS, the saturation solubility of FL in the presence of various surfactants and oils was evaluated (see Table 3). The solubility test was performed at 60 °C to melt the surfactant in a solid state at room temperature and to get the maximum solubility of FL. Instead of using weight per unit volume, the measured solubility values of FL in the presence of surfactants or oils were expressed as a mass fraction (weight/weight percent), since this approach is more reasonable in terms of weighing the surfactant and oil agents in solid or highly viscous liquid states at room temperature by the scale (rather than measuring by volume). According to the results, the solubility of FL in the amphiphilic surfactant (30~35%) was higher than that in oil (20~25%), as FL also has amphiphilic-like properties with its structure moieties, i.e., a lipophilic benzene ring and a hydrophilic carboxyl group [20]. As shown in Table 3, the solubility values of FL in surfactants or oils were above 20% (*w*/*w*), i.e., significantly higher than those of raw FL in water without the studied agents, known to be 0.0005% (5 μg/mL) [3]. The highest solubility of FL was found to be 24.85 ± 0.20% and 34.28 ± 4.26%, in Kollisolv MCT 70 and TPGS, respectively. However, as shown by the results of one-way ANOVA, no statistically significant differences were observed in solubility values among oils or surfactants. Therefore, various vehicle compositions were characterized for the physicochemical properties and vehicle performances in further research.

### 3.2. Characterization of Various Vehicle Compositions

The weight ratio of oil to surfactant (i.e., 1/3, 2/2, or 3/1) was used to prepare vehicle mixtures; in the next step, these mixtures were evaluated for the particle size of the dispersed phase and the melting point (see Table 1). With an increase in the ratio of oil to surfactant, the mean particle size of the dispersed phase increased, and the melting point of vehicle decreased. These findings are consistent with previous reports [21]. The vehicle with Kollisolv MCT 70 showed a small particle size (132.1 ± 10.5 nm) and existed in the solid state with the melting point above 30 °C. The mean particle size of the vehicle containing Capmul MCM C8 was smaller than that of Lauroglycol 90 and Kollisolv MCT 70, but it existed in the liquid state at room temperature. The vehicle with the surfactant Gelucire 44/14 had a large particle size, but existed in the solid state at room temperature. On the other hand, the mean particle size of the vehicle made of the surfactant Kolliphor HS 15 was small when the oil component (Kollisolv MCT 70, Lauroglycol 90, or Capmul MCM C8) was included with a smaller or equal amount in the weight ratio (oil/Kolliphor HS = 1/3 or 2/2). Vehicles containing Kolliphor HS 15 existed mainly in the liquid state at room temperature, except for the vehicle composition of 1/3 weight ratio (Kollisolv MCT 70 over Kolliphor HS 15) as a solid. Finally, after the repeated experiments, vehicles composition of Kollisolv MCT 70 and TPGS at the weight ratio of 1/3, 2/2, or 3/1 yielded small and uniform particle size (132.12 ± 10.51 nm, 278.95 ± 26.25 nm, and 396.63 ± 21.34 nm, respectively), and existed in the solid state with high melting points (36.96 °C, 36.58 °C, 36.37 °C, respectively). Therefore, Kollisolv MCT 70 and TPGS were selected as the proper type of oil and surfactant for further research.

### 3.3. Construction of Pseudo-Ternary Phase Diagram

A total of 22 formulations were prepared, based on the FL solubility curve that was drawn using the measured solubility values. According to the observed physical states and the particle size of the dispersed phase, a particular pseudo-ternary phase diagram was constructed (see Figure 1). The calculated solubility curve of FL was considered to be a useful criterion in determining the maximum solubilization (%) of FL in each type of FL formulation. FL was assumed as completely dissolved in the compositions within the calculated solubility curve. The physical states of the SNEDDS preconcentrate were solids at room temperature when the weight ratios of FL and TPGS were either 10% and >50%, or 20% and 80%. In other regions, the SNEDDS preconcentrates existed in the semisolid and liquid states. With an increase the weight ratio of FL from 10% to 20%, the physical states changed from solid to liquid. When the weight ratio of FL became 20% or larger, solidification of TPGS was suppressed, possibly resulting from the high contents of FL. The particles in the dispersed phase showed small sizes of below 100 nm in the formulations of FL = 10% and TPGS of ≥60%. Therefore, the pseudo-ternary phase diagram constructed by compositions of drug (FL), oil, and surfactant was regarded as a useful tool to confirm the effect of the drug contents on particle sizes in the dispersed phase, and to set the weight % range of vehicle compositions for solubilizing water-insoluble drugs. Based on the phase diagram (see Figure 1), we organized important findings such as physical states, particle sizes, and peak melting points for the representative SSP formulations composed of FL, Kollisolv MCT 70, and TPGS with various weight ratios (see Table 2).

### 3.4. Particle Size and Peak Melting Temperature

Size range of particles in the SNEDDS is an essential factor that could affect drug dissolution kinetics and bioavailability [22]. According to numerous previous reports, nano-sized particles can possess a large surface area, which contributes to achieving a rapid dissolution and absorption enhancement [23]. In Table 2, F1~F4 containing 10% FL exhibited very small and uniform particle sizes of below 50 nm with polydispersity index (PI) less than 0.3 (PI; F1 = 0.21 ± 0.05, F2 = 0.08 ± 0.03, F3 = 0.11 ± 0.05, F4 = 0.26 ± 0.11, mean ± S.D.). F5 showed the highest particle size on average, presumably due to the inclusion of Kollisolv MCT 70 with a high percentage (70%). Moreover, F5 produced precipitation of FL after storage of 4 weeks at room temperature (data not shown), which implicate the instability of liquid SNEDDS preconcentrate. But F1~F4, solid SNEDDS preconcentrates, gave good dispersion in water with no precipitation at that time point. F6~F8 containing 20% or 30% FL showed bigger particle sizes of 100~300 nm as compared to those of F1~F4. With an increase of the contents (%) of FL and a decrease of surfactants, the mass and volume of the dispersed FL could increase, thereby promoting an increase of particle sizes of dispersed droplets [24]. Peak melting point for F1~F4 was on average 30.74 °C~33.59 °C, which might have resulted from the high percentages of TPGS. However, F5~F9 containing FL > 20% or a low amount of TPGS (20%) exhibited no significant melting or low melting point of ≤30 °C with liquid or semi-solid states. As a result, F2 (FL/Kollisolv MCT 70/TPGS = 10/10/80, weight %) represented a small particle size of 13.74 ± 2.21 nm with a suitable solid state at room temperature.

### 3.5. Differential Scanning Calorimetry (DSC)

**Figure 2:**
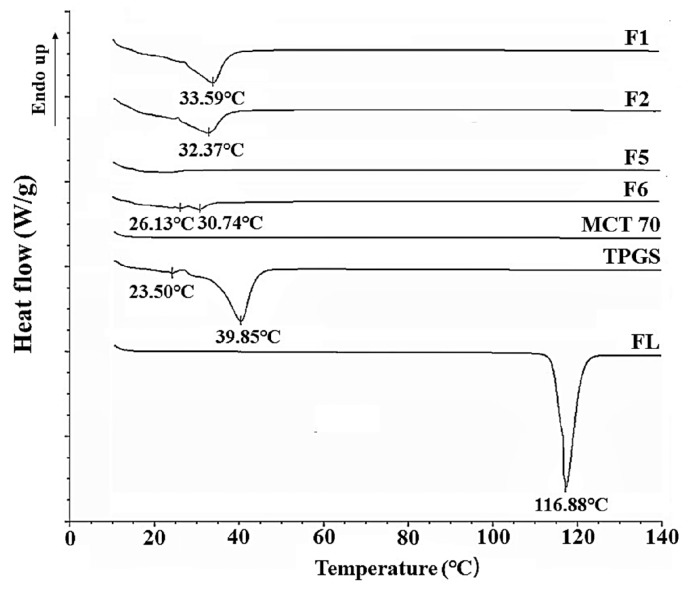
Differential scanning calorimetry (DSC) thermograms of F1, F2, F5, F6, Kollisolv MCT 70, TPGS and FL.

The thermal changes of the formulations were confirmed by the results of the DSC analysis. TPGS has been known to be a thermally stable surfactant with low melting and high decomposition temperatures and can be mixed with drug molecules to improve their physicochemical stabilities [25]. In our results (see Table 2), F1, F2, and F6 were found to be in the solid state at room temperature, and F5 was in the liquid state. Corresponding thermograms are shown in Figure 2. F1, F2, and F6 showed a newly-shifted single endothermic peak melting point (33.59 °C, 32.37 °C, 30.74 °C, respectively) probably derived from TPGS (39.85 °C) (see Figure 2). In F1, F2, F5, and F6, FL was completely dissolved, and the endothermic peak of the inherent melting point (116.88 °C) of raw FL disappeared, due to the amorphous state. With an increase of the weight ratio of TPGS in formulation, the peak melting temperature shifted to a lower temperature (see Figure 2). Compared to F1 containing 90% TPGS, F2 and F6 with 80% TPGS showed lower melting points. There were double peaks for F6 (20% FL), indicating a heterogeneous solid state (see Figure 2). Finally, F5 in the liquid state has shown no distinct endothermic peaks, suggesting that the formulation does not contain any crystalline forms (see Figure 2).

### 3.6. Powder X-ray Diffraction (PXRD)

**Figure 3:**
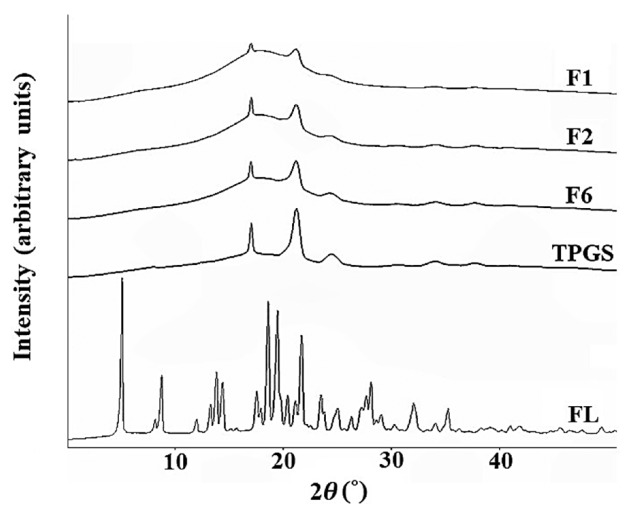
Powder X-ray diffraction (PXRD) curve of F1, F2, F6, TPGS and FL.

The PXRD patterns were analyzed for F1, F2, F6, TPGS and raw flurbiprofen (see Figure 3). The distinct peaks of TPGS (17.44°, 21.94°, and 24.80°) were observed in all three formulations (F1, F2, and F6), whereas the intrinsic peak of FL disappeared. This result suggests that FL might have been completely dissolved at the molecular level in the SNEDDS containing oil and surfactant. The intensity of the peaks derived from TPGS decreased in the formulations of F1, F2, F6. These PXRD patterns suggest that these formulations were partial mixtures of crystal and amorphous forms. With lower intensities than raw TPGS, the peaks from TPGS still remained in these formulations. This result can be attributed to the fact that TPGS partially existed in the crystalline state in those formulations, which were in the solid state at room temperature.

### 3.7. Dissolution Test

**Figure 4:**
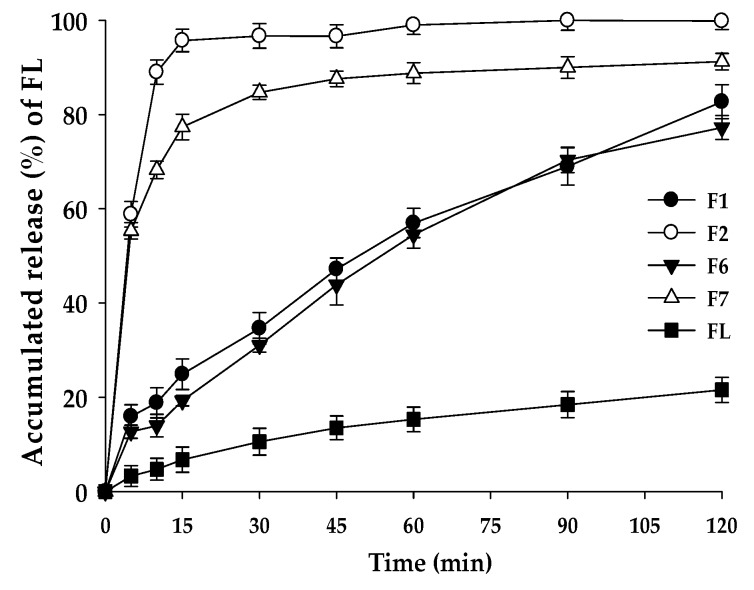
Dissolution profiles of F1, F2, F6, F7, and raw FL in pH 1.2 buffer. Values are represented as means ± SD (*n* = 3).

The dissolution test was performed for the solid formulations with the confirmed physical properties, including melting points and crystallinity (see Figure 4). The comparison of F2 and F7 suggests that the contents of FL (%) could have influenced the dissolution rate and the cumulative drug release level. F2 composed of FL/Kollisolv MCT 70/TPGS (10/10/80%) showed a rapid dissolution rate at pH 1.2, reaching the mean dissolution rate of 95.70% in 15 min. However, F1 composed of FL/Kollisolv MCT 70/TPGS with the ratio of 10/0/90% gave a slower mean dissolution rate with 24.91% in 15 min compared to that of F2. F7 with more Kollisolv MCT 70 (10%) provided a higher dissolution rate than F6 without this oil type. In F6 and F7, where commonly 20% FL was loaded, dispersed particle sizes were similar, but the mean dissolution rates in 15 min appeared very different (19.26% and 77.35%, respectively). This dramatic difference in dissolution profiles of F6 and F7 might be linked to whether Kollisolv MCT 70 was used. Based on these observations, Kollisolv MCT 70 was found to be an important component to facilitate FL release from the formulation. Previous studies on the emulsion formulations using Kollisolv MCT 70 support this conclusion [26]. This oil type can stabilize the core of the dispersed particles and help to spontaneously form drug dispersion [27].

### 3.8. Oral Pharmacokinetics

**Figure 5:**
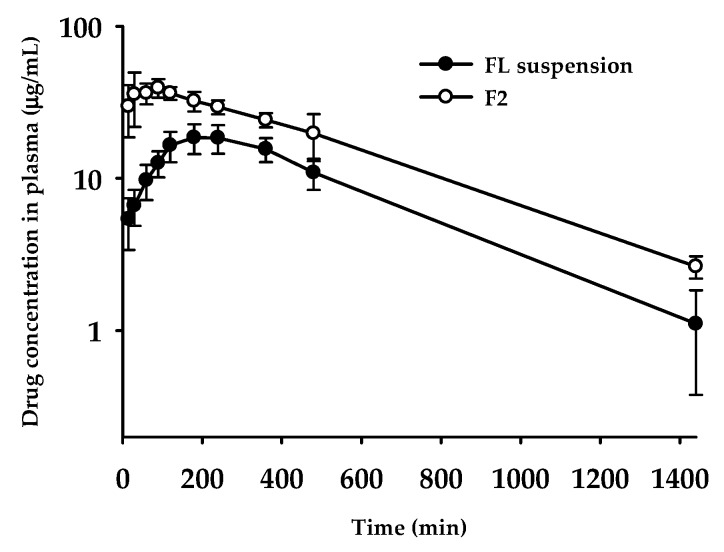
Plasma concentration–time profiles of FL after oral administration of FL suspension in 0.3% CMC-Na and F2 formulation at the dose of 10 mg/kg in rats. Each point represents the mean ± standard error (*n* = 4–5).

Based on the dissolution profile, F2 formulation containing FL was evaluated for oral pharmacokinetic studies in rats. Until 24 h after the administration of F2 formulation, whole plasma concentration profiles were compared with FL suspension in 0.3% CMC-Na. The calculated pharmacokinetic parameters are summarized in Table 4. As shown in Figure 5, as compared to the FL suspension group, the F2 formulation group maintained dramatically higher plasma concentrations. Maximum concentration in plasma (*C*_max_) and oral systemic exposure (i.e., AUC) were significantly increased in the F2 formulation group (*p* = 0.00002 for *C*_max_ and *p* = 0.00012 for AUC_∞_); 2.3-fold and 1.9-fold higher than FL suspension, respectively (see Table 4). As compared to the FL suspension group, the calculated relative bioavailability for F2 was observed to amount to 193%. The peak concentration time (*T*_max_) also changed significantly: From 228 min to 60 min. This behavior with F2 group reaching the maximum plasma concentration within a shorter time could be explained by dissolution profiles in vitro, showing much faster dissolution than FL suspension (see Figure 4). However, other pharmacokinetic parameters, such as half-life (*T*_1/2_) and MRT, remained unchanged, which means that, by contrast to the changed absorption phase, changing drug formulation did not affect the elimination phase. According to the correlation between dissolution profiles and pharmacokinetic data, enhanced solubility and dissolution rates in the F2 formulation possibly led to an improvement of oral absorption of FL in the gastrointestinal tract.

The pharmacokinetic results outlined above demonstrate that our strategy to develop a novel SNEDDS containing FL using surfactants or oils selected by the pseudo-ternary phase diagram has successfully contributed to optimizing the final SSP formulation. Initially, the SSP formulations produced by combinations of candidate vehicle compositions were screened for the physical states, particle sizes, and melting temperature, and then used for the construction of the phase diagram. After investigating dissolution kinetics, F2 formulation was determined as the optimal formula of FL, showing an enhanced solubility of FL in the physiological medium. Finally, the results of the in vivo oral absorption study confirmed that F2 formulation as the optimized SSP improved the oral bioavailability, with a 1.9-fold higher AUC than that of the raw FL.

## 4. Conclusions

In the present study, a novel SSP formulation containing FL was constructed based on the phase diagram, and physical properties were evaluated by measuring particle sizes, melting points and crystallinity; the dissolution test was also performed. The systemic approach used for optimizing SNEDDS of FL consisted of screening candidate vehicle compositions based on the pseudo-ternary phase diagram and establishing SSP formulation based on the selected vehicle compositions. The FL-solubilized SSP in the mixture of the selected oil and surfactant existed as a solid at room temperature, with the particle diameter of 50 nm or less and high dispersibility in the aqueous medium. The results of the analysis of the FL dissolution performance data revealed that the SNEDDS efficiently improved solubilization of poorly water soluble FL, providing a significantly higher dissolution rate than that of raw FL. Furthermore, in the results of the analysis of the in vivo pharmacokinetic data with SSP formulation, oral bioavailability of FL in rats dramatically increased, with 193% of relative bioavailability, as compared to the raw FL suspension. These findings suggest that this solid SNEDDS formulation could ensure physical stability of the drug product over the conventional liquid or solid SNEDDS and, most importantly, facilitate the oral absorption of the lipophilic drugs. Ultimately, the approach to developing solid SNEDDS, demonstrated in the present study, could contribute to improving drug formulations, thereby enhancing the bioavailability of various lipophilic drug molecules.

## Figures and Tables

**Table 1:** Physical properties according to the vehicle compositions.

Composition (Weight Ratio)			Physical State at 25 °C	Particle Size (nm, mean ± S.D., *n* = 3)	Peak Melting Temperature (°C, *n* = 1)
Kollisolv MCT 70	Gelucire 44/14	1/3	Solid	354.42 ± 21.82	41.68
		2/2	Solid	445.73 ± 34.30	41.22
		3/1	Solid	571.91 ± 40.48	40.99
	Kolliphor HS 15	1/3	Solid	129.40 ± 2.31	27.31
		2/2	Liquid	196.35 ± 18.73	No peak
		3/1	Liquid	490.63 ± 63.16	No peak
	TPGS	1/3	Solid	132.12 ± 10.51	36.96
		2/2	Solid	278.95 ± 26.25	36.58
		3/1	Solid	396.63 ± 21.34	36.37
Lauroglycol 90	Gelucire 44/14	1/3	Solid	533.96 ± 78.33	35.55
		2/2	Solid	546.20 ± 50.48	34.92
		3/1	Solid	592.21 ± 68.34	32.66
	Kolliphor HS 15	1/3	Liquid	228.95 ± 16.85	No peak
		2/2	Liquid	475.82 ± 35.43	No peak
		3/1	Liquid	795.76 ± 104.22	No peak
	TPGS	1/3	Solid	262.11 ± 30.47	36.70
		2/2	Liquid	556.73 ± 45.61	No peak
		3/1	Liquid	598.68 ± 61.63	No peak
Capmul MCM C8	Gelucire 44/14	1/3	Solid	171.30 ± 27.38	33.28
		2/2	Liquid	280.14 ± 56.94	No peak
		3/1	Liquid	490.26 ± 62.36	No peak
	Kolliphor HS 15	1/3	Liquid	98.16 ± 2.63	No peak
		2/2	Liquid	134.21 ± 9.45	No peak
		3/1	Liquid	312.86 ± 53.72	No peak
	TPGS	1/3	Solid	133.47 ± 4.34	35.36
		2/2	Liquid	259.23 ± 9.10	No peak
		3/1	Liquid	421.85 ± 48.53	No peak

**Table 2:** Physical properties according to representative formulations.

Formulation	Composition (Weight %)			Particle Size (nm, mean ± S.D., *n* = 3)	Peak Melting Temperature (°C, *n* = 1)
	FL	Kollisolv MCT 70	TPGS		
F1	10	0	90	26.82 ± 2.25	33.59
F2	10	10	80	13.74 ± 2.21	32.37
F3	10	20	70	17.03 ± 2.31	31.52
F4	10	30	60	31.42 ± 5.29	30.74
F5	10	70	20	594.38 ± 113.15	Liquid
F6	20	0	80	190.46 ± 48.37	26.13/30.72
F7	20	10	70	186.81 ± 24.21	Semi-solid
F8	30	0	70	245.24 ± 67.09	Liquid
F9	30	10	60	817.13 ± 128.77	Liquid

**Table 3:** Solubility of FL in surfactants and oils at 60 °C.

Vehicle	* Solubility (*w*/*w*%)
**Oils**	
Kollisolv MCT 70	24.85 ± 0.20
Lauroglycol 90	22.43 ± 0.92
Capmul MCM C8	21.38 ± 0.95
**Solid or Semisolid Surfactants**	
Gelucire 44/14	33.09 ± 1.44
Kolliphor HS 15	31.97 ± 2.45
TPGS	34.28 ± 4.26

* Data are expressed as the mean ± standard deviation (*n* = 3).

**Table 4:** Pharmacokinetic parameters of FL after oral administration of FL suspension in 0.3% CMC-Na and F2 formulation, at a dose of 10 mg/kg in rats (*n* = 4–5).

Pharmacokinetic Parameters	FL Suspension	F2
*T*_max_ (min)	228 ± 89	60.0 ± 34.6*
*C*_max_ (μg /mL)	19.6 ± 3.8	44.1 ± 3.4
*T*_1/2_ (min)	282 ± 73	340 ± 49
AUC_last_ (μg∙min/mL)	12700 ± 2052	24200 ± 2886*
AUC_∞_ (μg∙min/mL)	13200 ± 2279	25500 ± 2531*
MRT ^a^ (min)	448 ± 76	462 ± 34
Relative BA ^b^ (%)	-	193

^a^ MRT = AUMC/AUC; ^b^ Relative bioavailability (BA, %) = AUC_inf,F2 formulation_/AUC_inf,FL suspension_ × 100; * *p* < 0.05, compared with FL suspension based on a *t*-test.
